# Race and socioeconomic status interact with HPV to influence survival disparities in oropharyngeal squamous cell carcinoma

**DOI:** 10.1002/cam4.5726

**Published:** 2023-02-27

**Authors:** Emily Z. Yan, Benjamin M. Wahle, Sean T. Massa, Paul Zolkind, Randal C. Paniello, Patrik Pipkorn, Ryan S. Jackson, Jason T. Rich, Sidharth V. Puram, Angela L. Mazul

**Affiliations:** ^1^ Department of Otolaryngology‐Head and Neck Surgery Washington University School of Medicine St. Louis Missouri USA; ^2^ Department of Otolaryngology‐Head and Neck Surgery Saint Louis University School of Medicine St. Louis Missouri USA; ^3^ Department of Genetics Washington University School of Medicine St. Louis Missouri USA; ^4^ Division of Public Health Sciences, Department of Surgery Washington University School of Medicine St. Louis Missouri USA

**Keywords:** head and neck cancer, health care disparities, HPV, oropharyngeal squamous cell carcinoma, race, socioeconomic status, survival

## Abstract

**Background:**

HPV‐related oropharyngeal squamous cell carcinoma (OPSCC) is associated with a favorable prognosis, yet patients of color and low socioeconomic status (SES) continue to experience inferior outcomes. We aim to understand how the emergence of HPV has impacted race and SES survival disparities in OPSCC.

**Methods:**

A retrospective cohort of 18,362 OPSCC cases from 2010 to 2017 was assembled using the SEER (Surveillance, Epidemiology, and End Results) database. Cox proportional regression and Fine and Gray regression models were used to calculate hazard ratios (HRs) adjusting for race, SES, age, subsite, stage, and treatment.

**Results:**

Black patients had lower overall survival than patients of other races in HPV‐positive and HPV‐negative OPSCC (HR 1.31, 95% CI 1.13–1.53 and HR 1.23, 95% CI 1.09–1.39, respectively). Higher SES was associated with improved survival in all patients. Race had a diminished association with survival among high SES patients. Low SES Black patients had considerably worse survival than low SES patients of other races.

**Conclusion:**

Race and SES interact variably across cohorts. High SES was protective of the negative effects of race, although there remains a disparity in outcomes among Black and non‐Black patients, even in high SES populations. The persistence of survival disparities suggests that the HPV epidemic has not improved outcomes equally across all demographic groups.

## Graphical abstract

The epidemic of HPV‐related oropharyngeal squamous cell carcinoma (OPSCC) has remarkably changed the landscape of head and neck squamous cell carcinoma with the potential to improve outcomes and decrease treatment morbidity uniformly across demographic groups. This study evaluated the combined influence of race and socioeconomic status (SES) on OPSCC survival stratified by HPV status. Survival disparities between patients of high and low SES census tracts and Black and White race remained despite the increase in HPV‐related disease. Black patients with OPSCC had lower survival than patients of other races regardless of HPV status. High SES was protective of the adverse effects of race, although there remains a disparity in outcomes among Black and non‐Black patients, even in high SES populations.

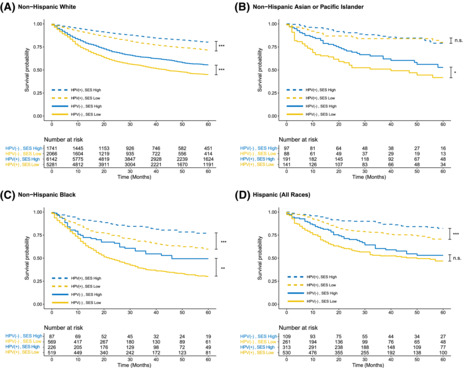

## 1 INTRODUCTION

Human papillomavirus (HPV) infection has been recognized as a significant risk factor for oropharyngeal squamous cell carcinoma (OPSCC), with about 60% of OPSCC cases in the US associated with HPV.^1^ Patients with HPV‐positive OPSCC are often younger, healthier, and more socioeconomically advantaged than those with HPV‐negative disease; they also demonstrate a dramatically improved prognosis.^2^, ^3^, ^4^ The epidemic of HPV‐related OPSCC has remarkably changed the landscape of head and neck squamous cell carcinoma with the potential to improve outcomes and decrease treatment morbidity uniformly across demographic groups.

Contemporary clinical trials routinely report 2‐year survival rates up to 95% despite using investigational de‐escalation protocols.^5^, ^6^ Meanwhile, many studies show inferior outcomes for oropharyngeal cancer patients among people of color and low socioeconomic status (SES) populations.^2^, ^7^, ^8^, ^9^ Controversy exists surrounding the relative contribution of race/SES in HPV‐related OPSCC survival disparities. Goodman et al. showed that Black patients with OPSCC had a 2.6‐fold greater risk of death than White patients after adjusting for HPV status.^10^ In contrast, other studies suggest that racial disparities in OPSCC are primarily related to HPV status given lower HPV prevalence among Blacks compared to Whites in OPSCC patients.^11^, ^12^, ^13^ Some studies even suggest no differences in survival by race in HPV‐positive cases; however, this has yet to be demonstrated at a population level.^14^ Finally, factors such as higher stage at diagnosis, comorbidities, SES, access to care, insurance status, and treatment differences are also potential contributors to survival disparities.^15^, ^16^, ^17^, ^18^, ^19^ Further work is needed to understand the relative contributions to these racial disparities in order to develop targeted public health interventions.

The overarching goal of this study was to understand how HPV has impacted race and SES survival disparities in OPSCC. We speculate that the intersection of race and neighborhood SES are critical prognostic factors even when stratifying by HPV status. We hypothesize that the survival disparities between patients of (1) high and low SES census tracts and (2) Black and White race remain despite the increase in HPV‐related disease.

## 2 METHODS

### 2.1 Study design and population

The SEER (Surveillance, Epidemiology, and End Results) 18 Census Tract‐level SES and Rurality Database (2000–2015) of the National Cancer Institute was utilized to assemble a retrospective cohort of OPSCC cases diagnosed between 2010 and 2017. OPSCC cases were defined using the following International Classification of Disease for Oncology (ICD‐O‐3) site codes: base of tongue (C01.9, C02.4), tonsil (C09.0, C09.1, C09.8, C09.9), and oropharynx (C05.1, C05.2, C10.0, C10.2, C10.3, C10.4, C10.8, C10.9). Cases were classified as HPV positive, HPV negative, or unknown/NA. HPV status was determined by positive in situ hybridization for high‐risk HPV (16, 18, 26, 31, 33, 35, 36, 45, 51–53, 56, 58, 59, 66–70, 73, 82, 85) and/or positive p16 staining by immunohistochemistry. Only cases with squamous cell histology were included (8052, 8083, 8078–8070).

### 2.2 Exposure assessment

Cases were grouped into HPV‐positive and HPV‐negative cohorts. Cases with unknown/NA HPV status were excluded in the survival analyses. The primary exposures of interest were race and SES. Other variables of interest included age, sex, year of diagnosis, primary subsite (base of tongue, tonsil, or other), stage, treatment, and rural–urban context. All covariate variables were available through SEER.

Race was defined as non‐Hispanic White, non‐Hispanic Black, non‐Hispanic Asian American or Pacific Islander, or Hispanic (all races). SES quintiles were available in SEER, with the first quintile representing the lowest SES group and the fifth quintile representing the highest SES group. These quintiles were generated using a SES index constructed from a factor analysis of seven census tract variables (median household income, median house value, median rent, percent below 150% of the poverty line, education index, percent working class, and percent unemployed) and linked to tumor cases at the census tract level based on diagnosis year.^20^ For some analyses in this study, SES was grouped as high (groups 4 and 5) and low (groups 1–3). Stage was defined as localized, regional, distant, or unknown using staging data from the SEER database. Treatment was defined as surgery (yes, no), chemotherapy (yes, no/unknown), and/or radiation (yes, no/unknown). The following surgical codes were included: 10, 12, 14, 20–28, 30–32, 40–43, 50–52, and 90. Rural–urban context was based on census tract‐level rurality variables using the US Department of Agriculture's Rural–Urban Communicating Area (RUCA) codes. RUCA was categorized using the Census Bureau's four‐category classification: All Rural, All Urban, Mostly Rural, and Mostly Urban. Rural–urban context was updated every 10 years.

### 2.3 Outcome assessment

The primary outcome was overall survival (OS), which was defined as duration from diagnosis date to death from any cause provided by SEER. The secondary outcome was cancer‐specific survival (CSS), which was defined as duration from diagnosis date to OPSCC‐related death. We censored patients after 5 years from diagnosis date.

### 2.4 Statistical analysis

Descriptive statistics were calculated with *t*‐tests, chi‐square, or exact tests where appropriate. *p* < 0.05 was considered statistically significant. Univariate survival analysis was performed with Kaplan–Meier curves and log‐rank tests. Restricted mean survival time (RMST) was estimated as an alternative measure of survival. RMST can be interpreted as the “life expectancy” between diagnosis and our chosen time horizon of 5 years, with the change in RMST (ΔRMST) representing the difference in “life expectancy” between two groups of interest.^21^ Multivariable Cox proportional regression models were used to calculate hazard ratios (HRs) for OS, adjusting for race, SES, age, subsite, stage, and treatment. Multivariable Fine and Gray regression models were used to calculate HRs for CSS with competing risks, adjusting for race, SES, age, subsite, stage, and treatment. R (4.0.3) was used to conduct all statistical analyses. The *survival* (3.2–7) and *survminer* (0.4.8) R packages were used for survival analysis.^22^, ^23^, ^24^

## 3 RESULTS

### 3.1 Descriptive statistics

In total, 18,362 OPSCC patients had HPV status and were included in the analysis (Table 1). A total of 13,343 (72.7%) represented HPV‐positive cases. HPV‐positive patients were more likely to be male than HPV‐negative patients (*n* = 11,552, 86.6% vs. *n* = 3782, 75.4%). The number of HPV‐positive OPSCC cases, as well as the proportion of OPSCC cases that were HPV‐positive, diagnosed each year trended upward from 2010 to 2017. HPV‐positive patients were more likely to be White than HPV‐negative patients (*n* = 11,423, 85.6% vs. *n* = 3807, 75.9%). Likewise, Hispanic, Asian American or Pacific Islander (AAPI), and Black patients comprised a larger proportion of HPV‐negative patients than HPV‐positive patients. We noted a trend of higher SES among HPV‐positive patients, with HPV‐positive patients more likely to be in the highest quintile for SES than their HPV‐negative counterparts (*n* = 3727, 27.9% vs. *n* = 1050, 20.9%).

**TABLE 1:** Descriptive statistics of OPSCC cases by HPV status.

	HPV‐negative	HPV‐positive	Unknown/NA	*p* value
*n*	(*N* = 5019)	(*N* = 13,343)	(*N* = 15,425)	
Primary site (%)				<0.001
Base of tongue	1945 (38.8)	5322 (39.9)	6379 (41.4)	
Tonsil	2029 (40.4)	7077 (53.0)	6281 (40.7)	
Other	1045 (20.8)	944 (7.1)	2765 (17.9)	
Sex (%)				<0.001
Male	3782 (75.4)	11,552 (86.6)	12,285 (79.6)	
Female	1237 (24.6)	1791 (13.4)	3140 (20.4)	
Year of diagnosis (%)				<0.001
2010	319 (6.4)	563 (4.2)	2726 (17.7)	
2011	539 (10.7)	866 (6.5)	2454 (15.9)	
2012	596 (11.9)	1241 (9.3)	2059 (13.3)	
2013	665 (13.2)	1618 (12.1)	1917 (12.4)	
2014	730 (14.5)	1878 (14.1)	1823 (11.8)	
2015	705 (14.0)	2099 (15.7)	1665 (10.8)	
2016	772 (15.4)	2472 (18.5)	1408 (9.1)	
2017	693 (13.8)	2606 (19.5)	1373 (8.9)	
Race/Ethnicity (%)				<0.001
Non‐Hispanic White	3807 (75.9)	11,423 (85.6)	12,050 (78.1)	
Hispanic (All races)	370 (7.4)	843 (6.3)	1222 (7.9)	
Non‐Hispanic AAPI	185 (3.7)	332 (2.5)	466 (3.0)	
Non‐Hispanic Black	657 (13.1)	745 (5.6)	1687 (10.9)	
SES (%)				<0.001
Group 1 (lowest SES)	1054 (21.0)	1581 (11.8)	2996 (19.4)	
Group 2	967 (19.3)	2138 (16.0)	3047 (19.8)	
Group 3	964 (19.2)	2752 (20.6)	3270 (21.2)	
Group 4	984 (19.6)	3145 (23.6)	3176 (20.6)	
Group 5 (highest SES)	1050 (20.9)	3727 (27.9)	2936 (19.0)	
RUCA (%)				<0.001
All Rural	345 (6.9)	871 (6.5)	1096 (7.1)	
All Urban	3288 (65.5)	8409 (63.0)	9814 (63.6)	
Mostly Rural	337 (6.7)	1028 (7.7)	1233 (8.0)	
Mostly Urban	1049 (20.9)	3035 (22.7)	3282 (21.3)	
Stage (%)				<0.001
Localized	840 (16.7)	1080 (8.1)	2330 (15.1)	
Regional	3049 (60.7)	10,048 (75.3)	9294 (60.3)	
Distant	1062 (21.2)	2081 (15.6)	2971 (19.3)	
Unknown/Unstaged	68 (1.4)	134 (1.0)	830 (5.4)	

Abbreviations: AAPI, Asian American or Pacific Islander; HPV, human papilloma virus; RUCA, rural urban commuting area; SES, socioeconomic status.

In the cohort, 22.3% of all Black patients were high SES, while 51.8% of all White patients were high SES (Table S1). HPV‐positive low SES Black patients were less likely to be treated with surgery than low SES patients of other races. Similarly, HPV‐negative high and low SES Black patients were less likely to be treated with surgery than their SES counterparts of other races.

### 3.2 Univariate survival

**FIGURE 1:**
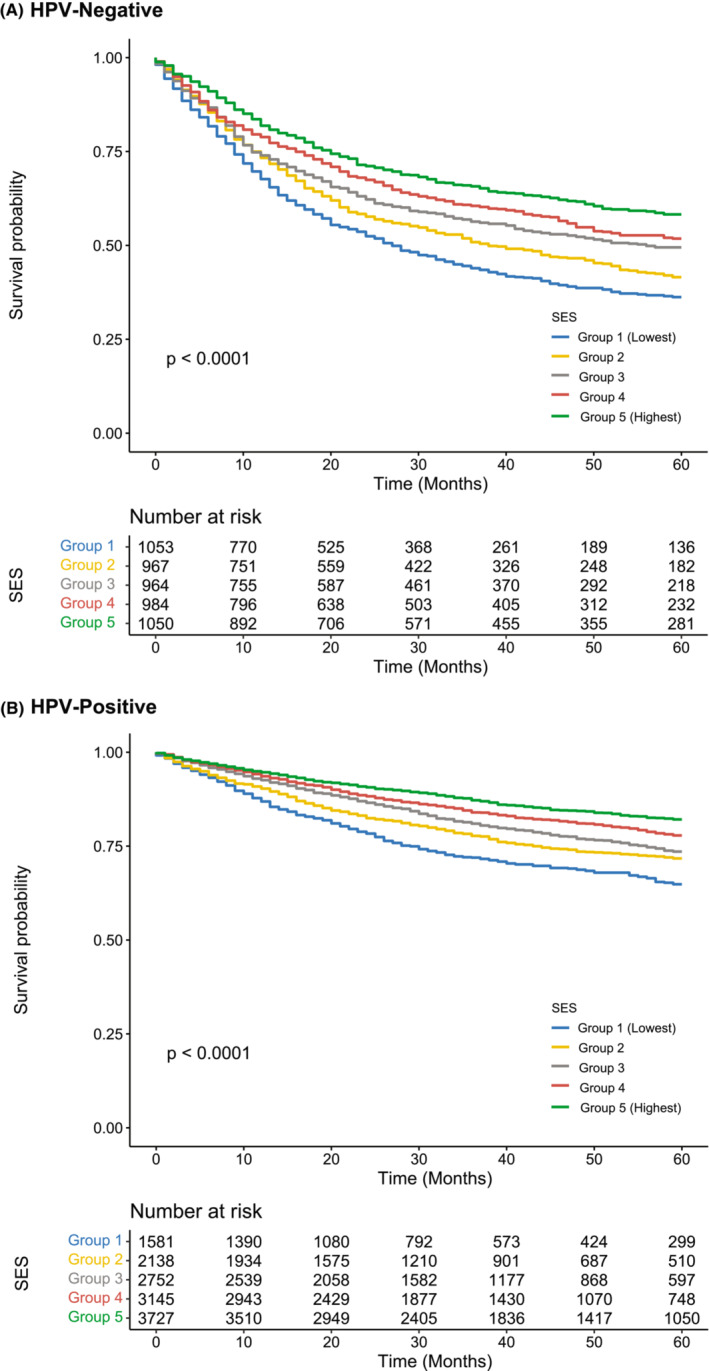
Survival outcomes by socioeconomic status (SES) in (A) HPV‐ and (B) HPV+ oropharyngeal squamous cell carcinoma. Living in low SES census tracts was associated with poor survival regardless of HPV status.

**FIGURE 2:**
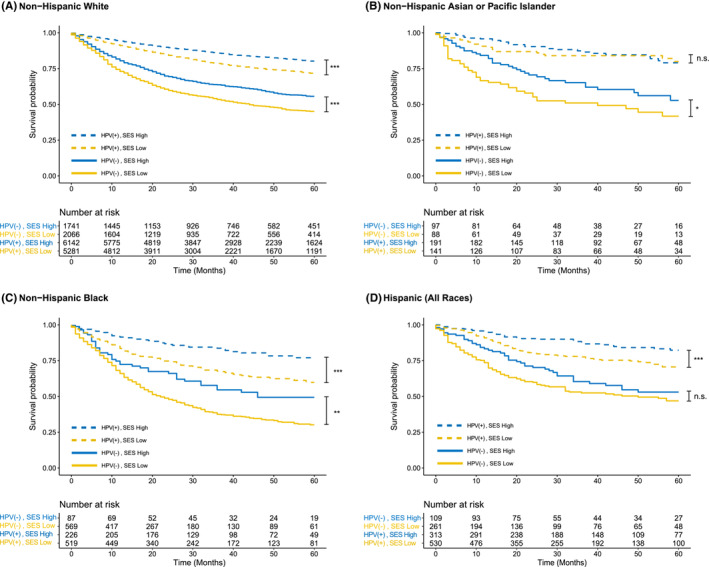
Survival outcomes by HPV and socioeconomic status (SES) in oropharyngeal squamous cell carcinoma (OPSCC) (*p < 0.05, **p < 0.01, ***p < 0.001, n.s. = not significant). (A) For White patients, low SES was associated with poor survival regardless of HPV status. (B) For Asian or Pacific Islander patients, SES was not associated with significant differences in survival in HPV‐positive OPSCC. (C) For Black patients, low SES was associated with poor survival regardless of HPV status. (D) For Hispanic patients, SES was not associated with significant differences in survival in HPV‐negative OPSCC.

Across OPSCC patients of all races, living in low SES census tracts was associated with poor survival regardless of HPV status (Figure 1). Among HPV‐positive patients, White, Black, and Hispanic patients of low SES displayed worse survival than their high SES counterparts (Figure 2). SES was not associated with significant differences in survival among AAPI patients with HPV‐positive OPSCC. Among HPV‐negative patients, White, AAPI, and Black patients of low SES displayed worse survival than their high SES counterparts. SES was not associated with significant differences in survival among Hispanic patients with HPV‐negative OPSCC.

While high SES was associated with improved survival for all races, both high and low SES Black patients experienced lower 5‐year OS than their respective SES counterparts of other races (Table S2). In HPV‐positive OPSCC, high SES Black and White patients had 5‐year OSs of 77.1% and 80.2%, respectively. Low SES Black and White patients had 5‐year OSs of 59.8% and 71.7%. Similarly, in HPV‐negative OPSCC, high SES Black and White patients had 5‐year OSs of 49.4% and 55.6%. Low SES Black and White patients had 5‐year OSs of 30.2% and 45.1%.

ΔRMSTs over a 5 year follow‐up period showed widening disparities in survival between HPV‐positive and HPV‐negative OPSCC (Table S2). In HPV‐positive OPSCC, low SES Black patients survived 9.0 months (95% CI: −11.1, −7.0) less than high SES White patients, while low SES White patients survived 3.3 months (95% CI: −4.1, −2.7) less than high SES White patients. Conversely, in HPV‐negative OPSCC, low SES Black patients survived 6.6 months (95% CI: −8.1, −5.2) less than high SES White patients, while low SES White patients survived 5.1 months (95% CI: −6.7, −3.7) less than high SES White patients.

### 3.3 Multivariable survival (OS)

Black patients consistently had worse survival than patients of other races in both HPV‐positive and HPV‐negative OPSCC (HR 1.31, 95% CI 1.13–1.53 and HR 1.23, 95% CI 1.09–1.39, respectively) (Table 2). Higher SES was associated with improved survival in all patients. In HPV‐positive OPSCC, low SES Hispanic, Black, and White but not AAPI patients displayed significantly worse survival than high SES White patients (Table 3). In HPV‐negative OPSCC, low SES Hispanic, AAPI, Black, and White patients displayed significantly worse survival than high SES White patients. Though not significant, high SES Black patients displayed a trend of worse survival than high SES White patients in both HPV‐positive and negative OPSCC. Across all races and regardless of HPV, low SES OPSCC patients had worse survival.

**TABLE 2:** Adjusted hazard ratios for overall survival from a multivariable Cox Proportional regression.

	HPV‐positive		HPV‐negative	
	Hazard ratio^a^ (95% CI)	*p* value	Hazard ratio^a^ (95% CI)	*p* value
Race/Ethnicity				
White	1	—	1	—
Hispanic (All Races)	1.01 (0.85, 1.19)	0.94	0.94 (0.79, 1.11)	0.45
Non‐Hispanic AAPI	0.9 (0.68, 1.19)	0.44	1.07 (0.86, 1.34)	0.56
Non‐Hispanic Black	1.31 (1.13, 1.53)	<0.01	1.23 (1.09, 1.39)	<0.01
SES				
Group 1 (lowest SES)	2.2 (1.92, 2.51)	<0.01	1.67 (1.45, 1.92)	<0.01
Group 2	1.72 (1.51, 1.95)	<0.01	1.47 (1.28, 1.69)	<0.01
Group 3	1.49 (1.32, 1.69)	<0.01	1.19 (1.04, 1.37)	0.01
Group 4	1.2 (1.06, 1.36)	<0.01	1.1 (0.95, 1.26)	0.20
Group 5	1	—	1	—

Abbreviations: HPV, human papilloma virus; CI, confidence interval; AAPI, Asian American or Pacific Islander; SES, socioeconomic status.

^a^ Adjusted for age at diagnosis, subsite, stage, chemotherapy, radiation, and surgery.

**TABLE 3:** Adjusted hazard ratios for overall survival for race*SES from a multivariable Cox Proportional regression.

		HPV‐positive				HPV‐negative			
		HR^a^ (95% CI)	*p*	HR^a^ (95% CI)	*p*	HR^a^ (95% CI)	*p*	HR^a^ (95% CI)	*p*
Race	SES								
White	High	1		1		1		1	
	Low	1.56 (1.43, 1.71)	<0.01	1.57 (1.43, 1.71)	<0.01	1.34 (1.22, 1.48)	<0.01	1.34 (1.21, 1.48)	<0.01
Hispanic (All Races)	High	0.95 (0.69, 1.31)	0.76	1		1.00 (0.74, 1.37)	0.98	1	
	Low	1.68 (1.38, 2.05)	<0.01	1.75 (1.21, 2.53)	<0.01	1.29 (1.06, 1.58)	0.01	1.27 (0.89, 1.83)	0.19
AAPI	High	1.00 (0.68, 1.46)	0.98	1		1.02 (0.73, 1.43)	0.90	1	
	Low	1.25 (0.82, 1.89)	0.30	1.22 (0.67, 2.20)	0.52	1.46 (1.08, 1.98)	0.01	1.43 (0.89, 2.29)	0.14
Black	High	1.31 (0.95, 1.81)	0.10	1		1.25 (0.9, 1.72)	0.18	1	
	Low	2.32 (1.96, 2.75)	<0.01	1.67 (1.16, 2.40)	0.01	1.82 (1.59, 2.08)	<0.01	1.44 (1.03, 2.01)	0.03

Abbreviations: AAPI, Asian American or Pacific Islander; CI, confidence interval; HPV, human papilloma virus; HR, hazard ratio; *p*, *p*‐value; SES, socioeconomic status.

^a^ Adjusted for age at diagnosis, subsite, stage, chemotherapy, radiation, and surgery.

### 3.4 Multivariable survival (CSS)

Similar trends were observed between CSS and OS (Table 4). In HPV‐positive OPSCC, low SES Hispanic, Black, and White but not AAPI patients displayed significantly worse survival than high SES White patients. In HPV‐negative OPSCC, low SES AAPI, Black, and White but not Hispanic patients demonstrated substantially worse survival than high SES White patients. However, there was no longer a trend of high SES Black patients displaying worse survival than high SES White patients. Similar to OS, low SES OPSCC patients had worse CSS.

**TABLE 4:** Adjusted hazard ratios for cancer specific survival for race* SES with competing risks from a multivariable Fine and Gray Regression.

		HPV‐positive				HPV‐negative			
		HR^a^ (95% CI)	*p*	HR^a^ (95% CI)	*p*	HR^a^ (95% CI)	*p*	HR^a^ (95% CI)	*p*
Race	SES								
White	High	1		1		1		1	
	Low	1.48 (1.33, 1.65)	<0.01	1.56 (1.43, 1.71)	<0.001	1.19 (1.06, 1.34)	<0.01	1.19 (1.06, 1.34)	<0.01
Hispanic (All Races)	High	1.20 (0.84, 1.70)	0.32	1		1.00 (0.71, 1.4)	0.99	1	
	Low	1.97 (1.57, 2.47)	<0.01	1.64 (1.09, 2.46)	0.02	1.15 (0.91, 1.46)	0.25	1.17 (0.79, 1.75)	0.44
AAPI	High	0.92 (0.56, 1.52)	0.75	1		0.96 (0.64, 1.42)	0.83	1	
	Low	1.14 (0.69, 1.89)	0.60	1.28 (0.58, 2.80)	0.54	1.87 (1.35, 2.58)	<0.01	1.76 (1.03, 2.99)	0.04
Black	High	1.12 (0.74, 1.68)	0.60	1		1.05 (0.7, 1.58)	0.80	1	
	Low	2.15 (1.78, 2.65)	<0.01	1.72 (1.10, 2.69)	0.02	1.55 (1.32, 1.82)	<0.01	1.55 (1.03, 2.32)	0.03

Abbreviations: AAPI, Asian American or Pacific Islander; CI, confidence interval; HPV, human papilloma virus; HR, hazard ratio; *p*, *p*‐value; SES, socioeconomic status.

^a^ Adjusted for age at diagnosis, subsite, stage, chemotherapy, radiation, and surgery.

## 4 DISCUSSION

Race and SES disparities are individually well documented in OPSCC, with Black patients and patients of low SES consistently displaying poor survival outcomes.^9^, ^10^, ^18^, ^19^, ^25^, ^26^, ^27^, ^28^ As the first study to investigate intersectional survival disparities of race and SES stratified by HPV status, we unmasked survival patterns that otherwise would not have been apparent. It is critical to consider HPV status in survival analysis due to its distinct disease process, approach to treatment, and prognosis.

Black patients experienced inferior survival compared to patients of other races in both HPV‐positive and HPV‐negative OPSCC, which challenges the idea that racial disparities in OPSCC are primarily related to HPV status, given lower HPV prevalence among Blacks than Whites.^11^, ^12^, ^13^ Our results suggest that other social and societal factors, including access to care, insurance status, treatment differences, social support, environmental exposures, or variation in tumor biology, may contribute to disparities in survival. Notably, Black individuals are more frequently uninsured and in poverty.^29^ In 2019, only 9% of White individuals were living in poverty, in stark contrast to 21% of Black individuals.^30^ Irrespective of SES or cancer stage, Black patients with OPSCC do not receive the same treatment recommendations as their White counterparts and less frequently receive surgery.^31^ Finally, Black and socioeconomically disadvantaged patients are less likely to have adequate follow‐up care for their cancers, as seen in breast and colorectal cancer.^32^, ^33^, ^34^

Race‐specific differences in tumor biology have been demonstrated in breast and prostate cancer, among others.^35^, ^36^ More recently, researchers have begun to explore the molecular biological characteristics of OPSCC. For example, silencing of tumor suppressor protein p16INK4 has been established as a negative prognosticator in OPSCC survival and occurs more often in Black patients compared to White patients.^16^ Koyuncu et al. also showed significant differences in tumor multinucleation, an adverse prognostic factor, between Black and White patients with HPV‐related OPSCC.^37^

Our findings highlight a dynamic interaction between race and SES in survival. Notably, low SES Black patients had considerably worse survival than low SES patients of other races. Although not significant, high SES Black patients also had lower OS than their high SES counterparts of other races. Given the small sample size of high SES Black patients, the study was not powered to detect a significant difference in survival. However, this trend has been similarly demonstrated in other cancers and supports the “diminishing returns hypothesis”—people of color are unable to attain the same health returns and benefits at high SES levels compared to their high SES counterparts of other races.^38^, ^39^ In our study, particular racial identities also had higher survival than expected for their SES. For example, SES was not associated with significant differences in survival outcomes among AAPI patients with HPV‐positive OPSCC, unlike in patients of other races. Immigrant populations have better cancer outcomes. Literature has suggested that living in ethnic enclaves has been associated with lower cancer mortality in Hispanic and Asian communities.^40^, ^41^

Differences in SES, access to care, and treatment inadequately explain why Black patients continue to have disparate health outcomes, not only in cancer but also in pregnancy, heart disease, diabetes, and obesity.^42^, ^43^, ^44^ It is essential to consider the contribution of allostatic load, the concept that the chronic stress associated with a lifetime of race‐related discrimination and inequality leads to physiologic dysregulation and increased risk for illness.^45^, ^46^ Disproportionate exposure to these stressors across a lifetime can help explain why Black and other minority groups experience worse longitudinal health outcomes.^42^, ^43^, ^44^ Allostatic load may also explain how even high SES, a protective factor for other races, fails to insulate Black patients against risk in OPSCC. Increasing education and upward mobility may amplify exposure to discrimination for minority individuals, leading to physiologic change.^47^

We noted that race had a diminished association with CSS compared to OS, particularly among high SES patients. Thus, high SES appears to be protective of the adverse effects of race. Du et al. similarly demonstrated that racial disparities in prostate carcinoma survival reduced substantially after controlling for socioeconomic factors, indicating that SES remains a significant barrier to achieving equal outcomes.^48^ The difference between OS and CSS models also suggests that Black patients frequently die of non‐cancer‐related causes. However, it is essential to consider these two models' different usages and interpret each in its specific context. CSS is beneficial when we expect the competing mortality to be substantial and distinct among the race/SES groups. Nonetheless, it does not account for cancer‐related treatment toxicities or exacerbation of comorbidities. This could result in death being documented as non‐cancer related and falsely improved CSS.

These findings provide important considerations for the de‐escalation of treatment in HPV‐positive OPSCC. First, low SES patients and patients of color may not qualify for de‐escalation due to higher stage at diagnosis, greater tobacco use history, and more comorbidities. Without including these patients in modern trials, it is uncertain whether they may be overtreated and suffer unnecessary toxic effects, or inappropriately de‐escalated as de‐escalation protocols become more commonly used. The poor survival observed here by SES and race suggests that targeting oncologic outcomes for these populations should be higher priority than addressing morbidity concerns among selected populations with excellent oncologic outcomes. Ultimately, the longstanding, unaddressed class‐based disparities in OPSCC outcomes will not be resolved by the improved prognosis of HPV‐related disease if the excellent clinical trial outcomes are not experienced broadly and equitably.

The limitations of this study are related to the use of cancer registry data. First, we acknowledge that the classification of stage as localized, regional, or distant is a significant limitation of our survival models. For instance, nodal/regional disease is not a significant prognostic indicator in HPV‐positive OPSCC.^49^ However, given the lack of complete AJCC staging data in the SEER database and the shift from 7th to 8th edition in 2018, this classification remains the best approximation for stage. Second, the coding of treatment modalities in the SEER database makes it difficult to determine treatment definitively. However, previous studies have shown that surgery with adjuvant chemotherapy/radiation therapy and definitive chemotherapy/radiation therapy have equivalent outcomes in OPSCC.^50^ As a result, we chose to include treatment in our models to explore its effect size on survival but recognize that the utility is limited. Third, the SEER database does not provide individual subject‐level SES data. Although census tract SES represents a patient's built environment, it may not capture individual‐level SES. Additionally, we lacked data on tobacco and alcohol use, a critical mediator that may differ by SES and race. Tobacco use is also an independent negative prognostic factor in OPSCC.^2^, ^51^, ^52^ Finally, we acknowledge that the small sample size of some populations, such as high SES Black patients, reduces the power to detect survival differences.

In conclusion, our study expands on the findings of previous studies by evaluating the collective role of race and SES on survival in OPSCC stratified by HPV status. We found that Black patients consistently had worse survival than patients of other races regardless of HPV status and that race and SES interact variably across cohorts. Most notably, high SES was largely protective of the adverse effects of race. However, there remains a disparity in outcomes among Black and non‐Black patients, even in high SES populations. Importantly, the persistence of survival disparities suggests that the HPV epidemic has not produced equivalent outcomes across all demographic groups. It is crucial to recognize that these survival disparities are mediated by complex interactions in the footprint of a long history of racism, including social, structural, and environmental pressures rather than individual patient factors and health behaviors.^42^ As treatment options for OPSCC continue to evolve, healthcare providers and policymakers must develop a greater understanding of the effects of racism, allostatic load, and access to care and their role in health outcomes.

## AUTHOR CONTRIBUTIONS

**Emily Z. Yan:** Conceptualization (equal); investigation (equal); visualization (equal); writing – original draft (lead); writing – review and editing (lead). **Benjamin M. Wahle:** Conceptualization (equal); visualization (equal); writing – review and editing (equal). **Sean T. Massa:** Conceptualization (equal); methodology (equal); writing – review and editing (equal). **Paul Zolkind:** Writing – review and editing (equal). **Randal C Paniello:** Writing – review and editing (equal). **Patrik Pipkorn:** Writing – review and editing (equal). **Ryan Jackson:** Writing – review and editing (equal). **Jason T Rich:** Writing – review and editing (equal). **Sid Puram:** Conceptualization (equal); methodology (equal); project administration (equal); supervision (equal); writing – review and editing (equal). **Angela Liu Mazul:** Conceptualization (equal); data curation (equal); formal analysis (equal); investigation (equal); methodology (equal); project administration (equal); supervision (equal); visualization (equal); writing – review and editing (equal).

## FUNDING INFORMATION

ALM is supported by the National Institute of Minority Health and Health Disparities (K01MD013897). BMW is supported by the National Institute of Deafness and Other Communication Disorders within the National Institutes of Health, through the “Development of Clinician/Researchers in Academic ENT” training grant (T32DC000022). ALM and SVP are supported by the Barnes Jewish Foundation. The content is solely the responsibility of the authors and does not represent the official views of the National Institutes of Health.

## CONFLICT OF INTEREST STATEMENT

None.

## Supporting information


Table S1–S2.

## Data Availability

The data analyzed in this work can be found in SEER, a publicly available database (https://seer.cancer.gov/data/index.html).

## References

[1] Marur S , D'Souza G , Westra WH , Forastiere AA . HPV‐associated head and neck cancer: a virus‐related cancer epidemic. Lancet Oncol. 2010;11(8):781‐789. doi:10.1016/S1470-2045(10)70017-6 20451455 PMC5242182

[2] Ang KK , Harris J , Wheeler R , et al. Human papillomavirus and survival of patients with oropharyngeal cancer. N Engl J Med. 2010;363(1):24‐35. doi:10.1056/NEJMOA0912217/SUPPL_FILE/NEJMOA0912217_DISCLOSURES.PDF 20530316 PMC2943767

[3] Rettig EM , D'Souza G . Epidemiology of head and neck cancer. Surg Oncol Clin N Am. 2015;24(3):379‐396. doi:10.1016/J.SOC.2015.03.001 25979389

[4] Chaturvedi AK , Engels EA , Pfeiffer RM , et al. Human papillomavirus and rising oropharyngeal cancer incidence in the United States. J Clin Oncol. 2011;29(32):4294‐4301. doi:10.1200/JCO.2011.36.4596 21969503 PMC3221528

[5] Ferris RL , Flamand Y , Weinstein GS , et al. Phase II randomized trial of transoral surgery and low‐dose intensity modulated radiation therapy in resectable p16+ locally advanced oropharynx cancer: an ECOG‐ACRIN cancer research group trial (E3311). J Clin Oncol. 2022;40(2):138‐149. doi:10.1200/JCO.21.01752 34699271 PMC8718241

[6] Yom SS , Torres‐Saavedra P , Caudell JJ , et al. Reduced‐dose radiation therapy for HPV‐associated oropharyngeal carcinoma (NRG oncology HN002). J Clin Oncol. 2021;39(9):956‐965. doi:10.1200/JCO.20.03128 33507809 PMC8078254

[7] Suzuki I , Cullen KJ , Mehra R , Bentzen S , Goloubeva OG . Racial disparities in outcome among head and neck cancer patients in the United States: An analysis using SEER‐Medicare linked database 2019;37(15_suppl):6051. doi:10.1200/JCO.2019.37.15_SUPPL.6051

[8] Zandberg DP , Liu S , Goloubeva O , et al. Oropharyngeal cancer as a driver of racial outcome disparities in squamous cell carcinoma of the head and neck: 10‐year experience at the University of Maryland Greenebaum Cancer Center. Head Neck. 2016;38(4):564‐572. doi:10.1002/HED.23933 25488341 PMC4461547

[9] McDonald JT , Johnson‐Obaseki S , Hwang E , Connell C , Corsten M . The relationship between survival and socio‐economic status for head and neck cancer in Canada. J Otolaryngol Head Neck Surg. 2014;43:1‐6. doi:10.1186/1916-0216-43-2/TABLES/4 24422754 PMC3896831

[10] Goodman MT , Saraiya M , Thompson TD , et al. Human papillomavirus genotype and oropharynx cancer survival in The United States of America. Eur J Cancer. 2015;51(18):2759‐2767. doi:10.1016/J.EJCA.2015.09.005 26602016 PMC4666760

[11] Settle K , Posner MR , Schumaker LM , et al. Racial survival disparity in head and neck cancer results from low prevalence of human papillomavirus infection in black oropharyngeal cancer patients. Cancer Prev Res (Phila). 2009;2(9):776‐781. doi:10.1158/1940-6207.CAPR-09-0149 19641042 PMC4459126

[12] Faraji F , Rettig EM , Tsai HL , et al. The prevalence of human papillomavirus in oropharyngeal cancer is increasing regardless of sex or race, and the influence of sex and race on survival is modified by human papillomavirus tumor status. Cancer. 2019;125(5):761‐769. doi:10.1002/CNCR.31841 30521092

[13] Chernock RD , Zhang Q , El‐Mofty SK , Thorstad WL , Lewis JS . Human papillomavirus‐related squamous cell carcinoma of the oropharynx: a comparative study in whites and African Americans. Arch Otolaryngol Head Neck Surg. 2011;137(2):163‐169. doi:10.1001/ARCHOTO.2010.246 21339403 PMC3863596

[14] Fakhry C , Westra WH , Wang SJ , et al. The prognostic role of sex, race, and human papillomavirus in oropharyngeal and nonoropharyngeal head and neck squamous cell cancer. Cancer. 2017;123(9):1566‐1575. doi:10.1002/CNCR.30353 28241096 PMC5788020

[15] Gillison ML , Restighini C . Anticipation of the impact of human papillomavirus on clinical decision making for the head and neck cancer patient. Hematol Oncol Clin North Am. 2015;29(6):1045‐1060. doi:10.1016/J.HOC.2015.08.003 26568547

[16] Isayeva T , Xu J , Dai Q , et al. African Americans with oropharyngeal carcinoma have significantly poorer outcomes despite similar rates of human papillomavirus‐mediated carcinogenesis. Hum Pathol. 2014;45(2):310‐319. doi:10.1016/J.HUMPATH.2013.09.006 24355195

[17] Conway DI , Petticrew M , Marlborough H , Berthiller J , Hashibe M , Macpherson LMD . Socioeconomic inequalities and oral cancer risk: a systematic review and meta‐analysis of case‐control studies. Int J Cancer. 2008;122(12):2811‐2819. doi:10.1002/IJC.23430 18351646

[18] Megwalu UC , Ma Y . Racial disparities in oropharyngeal cancer survival. Oral Oncol. 2017;65:33‐37. doi:10.1016/J.ORALONCOLOGY.2016.12.015 28109465

[19] Megwalu UC . Impact of county‐level socioeconomic status on oropharyngeal cancer survival in the United States. Otolaryngol Head Neck Surg. 2017;156(4):665‐670. doi:10.1177/0194599817691462 28195022

[20] Census Tract‐level SES and Rurality Database ‐ SEER*Stat. Accessed January 24, 2023. https://seer.cancer.gov/seerstat/databases/census‐tract/

[21] Han K , Jung I . Restricted mean survival time for survival analysis: a quick guide for clinical researchers. Korean J Radiol. 2022;23(5):495‐499. doi:10.3348/KJR.2022.0061 35506526 PMC9081686

[22] Therneau T . A package for survival analysis in R. R package version 3.5‐3. 2023. Accessed January 9, 2023. https://CRAN.R‐project.org/package=survival

[23] Therneau TM , Grambsch PM . Modeling Survival Data: Extending the Cox Model. Springer; 2000. doi:10.1007/978-1-4757-3294-8

[24] Kassambara A , Kosinski M , Biecek P . survminer: Drawing Survival Curves using “ggplot2.” R package version 0.4.9. 2021. Accessed January 9, 2023. https://cran.r‐project.org/web/packages/survminer/index.html

[25] Osazuwa‐Peters N , Massa ST , Christopher KM , Walker RJ , Varvares MA . Race and sex disparities in long‐term survival of oral and oropharyngeal cancer in the United States. J Cancer Res Clin Oncol. 2016;142(2):521‐528. doi:10.1007/S00432-015-2061-8/TABLES/3 26507889 PMC11819284

[26] Ragin CC , Langevin SM , Marzouk M , Grandis J , Taioli E . Determinants of head and neck cancer survival by race. Head Neck. 2011;33(8):1092‐1098. doi:10.1002/HED.21584 20967872 PMC3380362

[27] Lenze NR , Farquhar DR , Mazul AL , Masood MM , Zevallos JP . Racial disparities and human papillomavirus status in oropharyngeal cancer: a systematic review and meta‐analysis. Head Neck. 2019;41(1):256‐261. doi:10.1002/HED.25414 30561088

[28] Stein E , Lenze NR , Yarbrough WG , Hayes DN , Mazul A , Sheth S . Systematic review and meta‐analysis of racial survival disparities among oropharyngeal cancer cases by HPV status. Head Neck. 2020;42(10):2985‐3001. doi:10.1002/HED.26328 32632953

[29] Kirby JB , Kaneda T . Unhealthy and uninsured: exploring racial differences in health and health insurance coverage using a life table approach. Demography. 2010;47(4):1035‐1051. doi:10.1007/BF03213738 21308569 PMC3000037

[30] Poverty Rate by Race/Ethnicity | KFF . Accessed May 29, 2022. https://www.kff.org/other/state‐indicator/poverty‐rate‐by‐raceethnicity/?currentTimeframe=0&selectedRows=%7B%22wrapups%22:%7B%22united‐states%22:%7B%7D%7D%7D&sortModel=%7B%22colId%22:%22Location%22%22sort%22:%22asc%22%7D

[31] Weng Y , Korte JE . Racial disparities in being recommended to surgery for oral and oropharyngeal cancer in the United States. Community Dent Oral Epidemiol. 2012;40(1):80‐88. doi:10.1111/J.1600-0528.2011.00638.X 21883357

[32] DiMartino LD , Birken SA , Mayer DK . The relationship between cancer survivors' socioeconomic status and reports of follow‐up care discussions with providers. J Cancer Edu. 2016;32(4):749‐755. doi:10.1007/S13187-016-1024-3 PMC5035178 27006193

[33] Jones BA , Dailey A , Calvocoressi L , et al. Inadequate follow‐up of abnormal screening mammograms: findings from the race differences in screening mammography process study (United States). Cancer Causes Control. 2005;16(7):809‐821. doi:10.1007/S10552-005-2905-7 16132791

[34] Laiyemo AO , Doubeni C , Pinsky PF , et al. Race and colorectal cancer disparities: health‐care utilization vs different cancer susceptibilities. J Natl Cancer Inst. 2010;102(8):538‐546. doi:10.1093/JNCI/DJQ068 20357245 PMC2857802

[35] Howlader N , Cronin KA , Kurian AW , Andridge R . Differences in breast cancer survival by molecular subtypes in the United States. Cancer Epidemiol Biomarkers Prev. 2018;27(6):619‐626. doi:10.1158/1055-9965.EPI-17-0627/69929/AM/DIFFERENCES-IN-BREAST-CANCER-SURVIVAL-BY-MOLECULAR 29593010

[36] Khani F , Mosquera JM , Park K , et al. Evidence for molecular differences in prostate cancer between african American and Caucasian men. Clin Cancer Res. 2014;20(18):4925‐4934. doi:10.1158/1078-0432.CCR-13-2265/86132/AM/EVIDENCE-FOR-MOLECULAR-DIFFERENCES-IN-PROSTATE 25056375 PMC4167562

[37] Koyuncu CF , Nag R , Lu C , et al. Image Analysis Reveals Differences in Tumor Multinucleations in Black and White Patients with Human Papillomavirus‐Associated Oropharyngeal Squamous Cell Carcinoma. Cancer. 2022;128:3831‐3842. doi:10.1002/CNCR.34446 36066461 PMC9782693

[38] Kish JK , Yu M , Percy‐Laurry A , Altekruse SF . Racial and ethnic disparities in cancer survival by neighborhood socioeconomic status in surveillance, epidemiology, and end results (SEER) registries. JNCI Monogr. 2014;2014(49):236‐243. doi:10.1093/JNCIMONOGRAPHS/LGU020 PMC4841168 25417237

[39] Keegan THM , Kurian AW , Gali K , et al. Racial/ethnic and socioeconomic differences in short‐term breast cancer survival among women in an integrated health system. Am J Public Health. 2015;105(5):938‐946. doi:10.2105/AJPH.2014.302406 25790426 PMC4386534

[40] Shariff‐Marco S , Gomez SL , Canchola AJ , et al. Nativity, ethnic enclave residence, and breast cancer survival among Latinas: variations between California and Texas. Cancer. 2020;126(12):2849‐2858. doi:10.1002/CNCR.32845 32181892 PMC7245543

[41] von Behren J , Abrahão R , Goldberg D , Gomez SL , Setiawan VW , Cheng I . The influence of neighborhood socioeconomic status and ethnic enclave on endometrial cancer mortality among Hispanics and Asian Americans/Pacific islanders in California. Cancer Causes Control. 2018;29(9):875‐881. doi:10.1007/S10552-018-1063-7 30056614 PMC7466936

[42] Riggan KA , Gilbert A , Allyse MA . Acknowledging and addressing Allostatic load in pregnancy care. J Racial Ethn Health Disparities. 2020;8(1):69‐79. doi:10.1007/S40615-020-00757-Z 32383045 PMC7647942

[43] Obeng‐Gyasi S , Tarver W , Carlos RC , Andersen BL . Allostatic load: a framework to understand breast cancer outcomes in black women. NPJ Breast Cancer. 2021;7(1):1‐4. doi:10.1038/s41523-021-00309-6 34330927 PMC8324921

[44] Beckie TM . A systematic review of allostatic load, health, and health disparities. Biol Res Nurs. 2012;14(4):311‐346. doi:10.1177/1099800412455688 23007870

[45] Geronimus AT , Hicken M , Keene D , Bound J . “Weathering” and age patterns of allostatic load scores among blacks and whites in the United States. Am J Public Health. 2006;96(5):826‐833. doi:10.2105/AJPH.2004.060749 16380565 PMC1470581

[46] Seeman TE , Singer BH , Rowe JW , Horwitz RI , McEwen BS . Price of adaptation—allostatic load and its health consequences: MacArthur studies of successful aging. Arch Intern Med. 1997;157(19):2259‐2268. doi:10.1001/ARCHINTE.1997.00440400111013 9343003

[47] Colen CG , Ramey DM , Cooksey EC , Williams DR . Racial disparities in health among nonpoor African Americans and Hispanics: the role of acute and chronic discrimination. Soc Sci Med. 2018;199:167‐180. doi:10.1016/J.SOCSCIMED.2017.04.051 28571900 PMC5673593

[48] Du XL , Fang S , Coker AL , et al. Racial disparity and socioeconomic status in association with survival in older men with local/regional stage prostate carcinoma. Cancer. 2006;106(6):1276‐1285. doi:10.1002/CNCR.21732 16475208

[49] Keane FK , Chen YH , Neville BA , et al. Changing prognostic significance of tumor stage and nodal stage in patients with squamous cell carcinoma of the oropharynx in the human papillomavirus era. Cancer. 2015;121(15):2594‐2602. doi:10.1002/CNCR.29402 25873094

[50] Sinha P , Karadaghy OA , Doering MM , Tuuli MG , Jackson RS , Haughey BH . Survival for HPV‐positive oropharyngeal squamous cell carcinoma with surgical versus non‐surgical treatment approach: a systematic review and meta‐analysis. Oral Oncol. 2018;86:121‐131. doi:10.1016/J.ORALONCOLOGY.2018.09.018 30409292

[51] Gillison ML , Zhang Q , Jordan R , et al. Tobacco smoking and increased risk of death and progression for patients with p16‐positive and p16‐negative oropharyngeal cancer. J Clin Oncol. 2012;30(17):2102‐2111. doi:10.1200/JCO.2011.38.4099 22565003 PMC3397696

[52] Chen SY , Last A , Ettyreddy A , et al. 20 pack‐year smoking history as strongest smoking metric predictive of HPV‐positive oropharyngeal cancer outcomes. Am J Otolaryngol. 2021;42(3):102915. doi:10.1016/J.AMJOTO.2021.102915 33482566 PMC8096678

