# Supplementary material for: Race and socioeconomic status interact with HPV to influence survival disparities in oropharyngeal squamous cell carcinoma
Source: Cancer Med. 2023 Feb 27;12(8):9976–87. doi: 10.1002/cam4.5726 (PMC10166958; doi:10.1002/cam4.5726)
Supplement: Supplementary file 1 [file CAM4-12-9976-s001.docx]

Supplemental Table 1. Descriptive statistics by race and SES for HPV-positive and negative OPSCC cases

	HPV-Positive								
	White		Hispanic		AAPI		Black		
	High SES	Low SES	High SES	Low SES	High SES	Low SES	High SES	Low SES	
**n**	6142	5281	313	530	191	141	226	519	
**Primary Site (%)**									**<0.001**
Base of Tongue	2678 (43.6)	2052 (38.9)	103 (32.9)	150 (28.3)	56 (29.3)	40 (28.4)	84 (37.2)	159 (30.6)	
Other	374 (6.1)	390 (7.4)	21 (6.7)	49 (9.2)	9 (4.7)	17 (12.1)	13 (5.8)	71 (13.7)	
Tonsil	3090 (50.3)	2839 (53.8)	189 (60.4)	331 (62.5)	126 (66.0)	84 (59.6)	129 (57.1)	289 (55.7)	
**Sex = Male (%)**	5430 (88.4)	4523 (85.6)	255 (81.5)	443 (83.6)	160 (83.8)	107 (75.9)	201 (88.9)	433 (83.4)	**<0.001**
**Year of Diagnosis (%)**									0.149
2010	283 (4.6)	214 (4.1)	14 (4.5)	11 (2.1)	6 (3.1)	5 (3.5)	7 (3.1)	23 (4.4)	
2011	421 (6.9)	344 (6.5)	12 (3.8)	31 (5.8)	10 (5.2)	8 (5.7)	18 (8.0)	22 (4.2)	
2012	599 (9.8)	469 (8.9)	34 (10.9)	50 (9.4)	13 (6.8)	13 (9.2)	20 (8.8)	43 (8.3)	
2013	753 (12.3)	636 (12.0)	38 (12.1)	63 (11.9)	36 (18.8)	20 (14.2)	25 (11.1)	47 (9.1)	
2014	871 (14.2)	736 (13.9)	46 (14.7)	66 (12.5)	27 (14.1)	21 (14.9)	31 (13.7)	80 (15.4)	
2015	981 (16.0)	827 (15.7)	51 (16.3)	79 (14.9)	30 (15.7)	20 (14.2)	33 (14.6)	78 (15.0)	
2016	1086 (17.7)	1021 (19.3)	49 (15.7)	108 (20.4)	33 (17.3)	26 (18.4)	40 (17.7)	109 (21.0)	
2017	1148 (18.7)	1034 (19.6)	69 (22.0)	122 (23.0)	36 (18.8)	28 (19.9)	52 (23.0)	117 (22.5)	
**Race/Ethnicity (%)**									**<0.001**
Hispanic (All Races)	0 (0.0)	0 (0.0)	313 (100.0)	530 (100.0)	0 (0.0)	0 (0.0)	0 (0.0)	0 (0.0)	
Non-Hispanic AAPI	0 (0.0)	0 (0.0)	0 (0.0)	0 (0.0)	191 (100.0)	141 (100.0)	0 (0.0)	0 (0.0)	
Non-Hispanic Black	0 (0.0)	0 (0.0)	0 (0.0)	0 (0.0)	0 (0.0)	0 (0.0)	226 (100.0)	519 (100.0)	

Supplemental Table 2. Five year overall survival, restricted mean survival time over a five year follow-up period, and difference in restricted mean survival time by race and SES for HPV-negative and positive OPSC

		HPV-Positive						HPV-Negative					
		5-year OS (95% CI)	RMST	ΔRMST	P Value	Stratum-specific ΔRMST	P Value	5-year OS (95% CI)	RMST	ΔRMST	P Value	Stratum-specific ΔRMST	P Value
**Race**	**SES**												
White	High	80.2 (79.0, 81.5)	53.2 (52.7, 53.6)	Ref	--	Ref	--	55.6 (53.1, 58.3)	42.0 (40.9, 43.1)	Ref	--	Ref	--
	Low	71.7 (70.2, 73.2)	49.8 (49.2, 50.3)	-3.4 (-4.1, -2.7)	<0.001	-3.3 (-4.0, -2.7)	<0.001	45.1 (42.7, 47.5)	36.8 (35.8, 37.9)	-5.2 (-6.7, -3.6)	<0.001	-5.1 (-6.7, -3.6)	<0.001
Hispanic (All Ra	High	82.3 (77.2, 87.8)	53.9 (52.1, 55.7)	0.7 (-1.1, 2.6)	0.441	Ref	--	53.1 (43.4, 64.8)	41.6 (37.3, 45.9)	-0.4 (-4.9, 4.0)	0.849	Ref	--
	Low	70.6 (65.8, 75.8)	49.0 (47.3, 50.8)	-4.1 (-5.9, -2.3)	<0.001	-5.0 (-7.5, -2.4)	<0.001	46.9 (40.3, 54.6)	36.8 (33.7, 39.9)	-5.2 (-8.5, -1.9)	0.002	-5.0 (-10.3, 0.3)	0.067
AAPI	High	79.0 (71.8, 87.0)	53.7 (51.4, 55.9)	0.5 (-1.8, 2.8)	0.672	Ref	--	52.8 (41.6, 66.8)	42.2 (37.6, 46.)	0.2 (-4.6, 5.0)	0.93	Ref	--
	Low	79.9 (72.1, 88.5)	52.1 (49.1, 55.2)	-1.0 (-4.1, 2.1)	0.519	-1.5 (-5.4, 2.4)	0.445	417 (31.1, 55.9)	34.6 (29.3, 40.0)	-7.4 (-12.8, -1.9)	0.008	-7.9 (-15.0, -0.82)	0.029
Black	High	77.1 (70.6, 84.2)	51.5 (49.1, 53.9)	-1.6 (-4.1, 0.8)	0.191	Ref	--	49.4 (39.1, 62.3)	38.4 (33.4, 43.5)	-3.6 (-8.8, 1.6)	0.177	Ref	--
	Low	59.8 (547, 65.4)	44.1 (42.1, 46.1)	-9.0 (-11.1, -7.0)	<0.001	-7.3 (-10.5, -4.2)	<0.001	30.2 (26.0, 35.1)	35.4 (34.4, 36.3)	-6.6 (-8.1, -5.2)	<0.001	-8.2 (-13.6, -2.7)	0.003

Abbreviations: HPV, human papilloma virus; OS, overall survival; CI, confidence interval; RMST, restricted mean survival time; SES, socioeconomic status; AAPI, Asian American or Pacific Islander
